# Autonomous Multi-Rotor Aerial Platform for Air Pollution Monitoring

**DOI:** 10.3390/s22030860

**Published:** 2022-01-23

**Authors:** Alexandru Cozma, Adrian-Cosmin Firculescu, Dan Tudose, Laura Ruse

**Affiliations:** Computer Science and Engineering Department, Faculty of Automatic Control and Computers, University Politehnica of Bucharest, Splaiul Independenței 313, Sector 6, 060042 Bucharest, Romania; alecsandru.cozma@gmail.com (A.C.); adrian.firculescu@cti.pub.ro (A.-C.F.); dan.tudose@upb.ro (D.T.)

**Keywords:** air pollution, mobile sensing, UAV, multi-rotor, hexa-rotor, multi-copter, urban, drone, gases

## Abstract

During the last few years, scientists have become increasingly concerned about air quality. Particularly in large cities and industrialised areas, air quality is affected by pollution from natural and anthropogenic sources and this has a significant impact on human health. Continuous monitoring of air quality is an important step in investigating the causes and reducing pollution. In this paper, we propose a new autonomous multi-rotor aerial platform that can be used to perform real-time monitoring of air quality in large cities. The air quality monitoring system is able to cover large areas, with high spatial resolution, even above average buildings, while being relatively low cost. We evaluate the proposed system in several locations throughout a metropolitan city, during different seasons and generate fine-grained heat-maps that display the level of pollution of specific areas based on different altitudes.

## 1. Introduction

Pollution has gradually become an important factor that affects the lifestyle and health of individuals, increasing morbidity and mortality [1]. Such an effect springs from the industrial revolution which has led to higher needs of energy and, in order to obtain it, the excessive burning of fossil fuels. Therefore, as people became more aware of the threat that pollution poses on their health, new projects have enabled individuals to actively measure concentrations of various gaseous agents that pose risks to human health [2,3].

There are certain areas around the globe where pollution has increased dramatically due to intensive industrial activities or because of the effect that certain procedures used in quarries, mills, or processing factories have on the nearby environment, thus drastically affecting the quality of life in any nearby residential housings. According to IQAir [4], in 2020, the most polluted city in the world was Hotan, China, mostly because of the sandstorms that have been aggravated by climate change. PM2.5 (fine particulate matter) concentration in Hotan, in September 2021, was 15 times the maximum value recommended by the World Health Organisation (WHO) and the air pollution level is considered hazardous [5]. Another factor that affects air quality in China is the high concentration of smog that originates from the coal burning factories that fuels China’s highly industrialised economy, but affects the health of individuals living within the vicinity.

A study that analysed the risk factors of chronic bronchitis for inhabitants of heavily polluted areas found that residents who live in environments adjacent to a mining or industrialised area have a 35% risk of experiencing a chronic cough with sputum, compared to only 18% for those residents who do not live in such environments [6]. Another study conducted in the industrialised city of Sumgayit, Azerbaijan, addressed concerns that inhabitants of neighbouring settlements have an increased risk of developing cancer as a consequence of intense industrial activities that affect air quality [7]. There are also studies that demonstrate that long-term exposure to high air pollution levels (PM2.5, PM10, O_3_, NO_2_, SO_2_, and CO) increases the incidence of COVID-19, as well as the infection severity and mortality associated with this disease [8,9,10]. For example, it was estimated that an increase of PM2.5 concentration with one unit is associated with a 9% increase in COVID-19-related mortality [8].

Considering international concerns regarding pollution in industrialised areas and metropolitan cities, we need an easy way to measure air pollution at different altitudes, with high spatial resolution over large areas. This research domain is still in the early stage, considering the lack of sufficient data available in the current literature [11]. With advancements in technology, sensing for gaseous pollutants has become less expensive [12]. This allows the development of new ways of measuring concentrations of various agents using specially designed portable devices. These can be used for collecting significant air quality data streams to be studied, therefore offering a new way of detecting low air quality environments.

In this paper, we propose a new UAV-based air quality real-time monitoring system equipped with a set of gas sensors, in order to investigate the air pollution levels in large cities. This method provides insight regarding how gaseous concentration disperses with altitude. The proposed system is able to cover large areas and high altitudes, even above normal buildings, while being low cost compared to aircraft-based solutions. We evaluate our system in several parts of the biggest city in Romania, in different seasons, by measuring air pollution at low and high altitudes. We generate fine-grained pollution dispersion maps for selected locations.

The rest of this article is structured as follows: The second section presents the state of the art solutions, while the third section provides a complete description of the proposed sensing system, with a thorough analysis of the major components. The fourth section focuses on interpreting the data that were gathered during the evaluation of the system. The fifth section presents the conclusions and contributions of the paper.

## 2. State of the Art

### 2.1. Micro Aerial Vehicles

Throughout the last decade, new developments in various engineering fields that involve high density power storage, integrated miniature electric actuators, or other micro electromechanical system (MEMS) technologies have opened up new possibilities that have made autonomous miniaturised flying robots a reality [13]. Although various unmanned aircrafts have existed, either non-motorised, such as a balloon or glider, or motorised, such as a miniaturised single propeller rotorcraft, each design has posed important drawbacks for reliable use in remote sensing.

Another type of unmanned motorised aircraft is a micro vertical take-off and landing (VTOL) rotorcraft, which is a specially designed aircraft system that emerges as a small-scale equivalent of a full-sized helicopter, more commonly referred to as a multi-rotor. As a new type of micro aerial vehicle (MAV), a multi-rotor air platform is rotor-based, most commonly integrating a four-rotor design that offers more manoeuvrability and reliability than a traditional single-rotor helicopter design. With the four rotors generally connected in an “X”, “+”, or “H” configuration, the altitude and direction are adjusted by varying the speed of individual propellers.

Even though the system has a relatively simple design, the reality shows that controlling quad-rotors pose difficult challenges and the platform can very easily lose balance and overturn. In order to prevent this, the rotors must react very quickly to counteract the tendency to tip over by adjusting the propeller speed accordingly. This must be done by using separate sensors such as accelerometers and gyroscopes that detect multi-rotor behaviour and offer a reference for central processing in order for it to quickly calculate and adjust the propeller speed before the system loses balance. This entire process happens hundreds of times within a second and requires high-level computation. Due to the advancements of electronics, the availability of low-cost processing units and motors that could update at such high rates was made possible [14].

The first possible small-scale unmanned VTOL (vertical take-off and landing) quad-rotor aircraft was developed at Stanford as the STARMAC project, which initially started as an automation project for the already commercially available Draganflyer X4 quad-rotor [15] and eventually led to the development of Stanford-created series of quadrotors, which improved the stability and the control of the design [16], thus allowing other applications or improvements to emerge.

Multiple types of MAVs have been used in various fields of study where certain advanced autonomous missions could not have been possible before, such as land and ocean remote sensing research [17,18,19] or studies that needed to be conducted in hostile environments, such as volcanic observations [20]. Nonetheless, MAVs offer a cost-effective solution to any research that requires spatial mobility or involves other autonomous missions such as long-term surveillance or search and rescue.

### 2.2. Pollution Tracking Using Unmanned Aerial Vehicles

In the last few years, the topic of air pollution monitoring using UAVs has been the attention of researchers and several solutions have been proposed. Pochwała et al. [21] use a multisensory array that is mounted on a DJI Matrice 200 drone and is able to measure several air pollutants at the same time: Ammonia (NH_3_), hexane (C_6_H_14_), benzene (C_6_H_6_), carbon monoxide (CO), carbon dioxide (CO_2_), hydrogen (H), methane (CH_4_), natural gas, liquefied petroleum gas (LPG), and suspended dust (PM2.5 and PM10). Their experiments show that the concentration of PM may increase with altitude and is correlated with the outside temperature. However, they do not analyse how other pollutants vary with height and they measure PM only up to 100 m in altitude.

Liu et al. [22] use a hexacopter equipped with small sensors to investigate the vertical profile of PM2.5 and black carbon (BC), up to 500 m above ground level, in Macau, China, in February and March. They concluded that PM2.5 concentrations decrease with height, with approximatively 0.2 $\mu g/m^{3}$ per 10 m. BC concentration has shown diverse vertical profiles and also has a vertical decrease of 0.1 $\mu g/m^{3}$ per 10 m. They also claim that wind, atmospheric stability, relative humidity, local emissions, and regional transport have a certain influence on the vertical profiles of these pollutants. They did not integrate other pollution sensors, as this remains to be for future work.

Rohi et al. [23] designed an aerial robotic system, called Environmental drone (E-drone), in order to obtain information about the weather and pollution. It is able to measure PM, CO_2_, CO, NH_3_, sulfur dioxide (SO_2_), ozone (O_3_), and nitrogen dioxide (NO_2_) concentrations and detect if they are higher than a certain limit. The E-drone has been tested in five locations and generated Air Quality Health Index (AQHI) maps. The novelty of the solution includes the ability to autonomously monitor air quality and provide pollution abatement for NO_2_. However, they do not provide abatement for O_3_, CO, CO_2_, SO_2_, NH_3_, and PM yet.

Gu and Jia [24] developed a UAV-based air quality monitoring system by using consumer components. The system includes a hexacopter and sensors for PM2.5 and NO_2_. They evaluated the proposed solution near a local road and near a highway with considerable traffic volume. They discovered that, because the UAV and on-board devices share the same power source, the ripple in the UAV’s current introduces noise into the sensors and data acquisition modules, which leads to modified sensor readings. The PM sensor is more noise-resistant than the NO_2_ sensor because it is based on an optical mechanism and includes its own data acquisition module. The PM sensor values are correlated with the ones published by the near-road monitoring stations. However, they do not provide a solution for isolating the sensors from the UAV’s interference and do not produce the vertical profile of the pollutants. In contrast, our proposed solution uses separate power sources for the UAV flying system and for the sensing unit.

Lambey and Prasad [25] wrote an extensive survey, in which they present the types of air quality sensors for CO, SO_2_, NO_2_, O_3_, PM2.5, PM10, and BC. They also describe a large number of UAV-based solutions for air quality monitoring. They identify the challenges regarding UAV-based systems, such as: Power consumption, weather conditions, equipment cost, and size. They conclude that UAVs represent a promising solution for air quality monitoring.

Our proposed solution uses low-cost, off-the-shelf sensors, and components in order to perform real-time monitoring of pollutants at low and high altitudes. It also uses separate power sources for the UAV flying system and for the sensing unit, so the sensor readings will not be affected by interference from the multi-copter. The system is able to cover large areas and high altitudes, while being low cost, and provides insight regarding how gaseous concentration disperses with altitude. We also use our solution to generate fine-grained pollution dispersion maps for the selected locations.

## 3. Architecture

In this paper, we propose a UAV-based system for real-time monitoring of pollution levels in metropolitan areas. The system combines a multi-gaseous sensing unit with an unmanned air vehicle that provides high spatial resolution over large areas at much lower costs than other aircraft surveillance. By making use of an accurate measurement system specifically designed for creating a detailed report of the flow of air pollutants, we can generate fine-grained pollution dispersion maps for specific areas.

### 3.1. Sensing Unit

**Figure 1:**
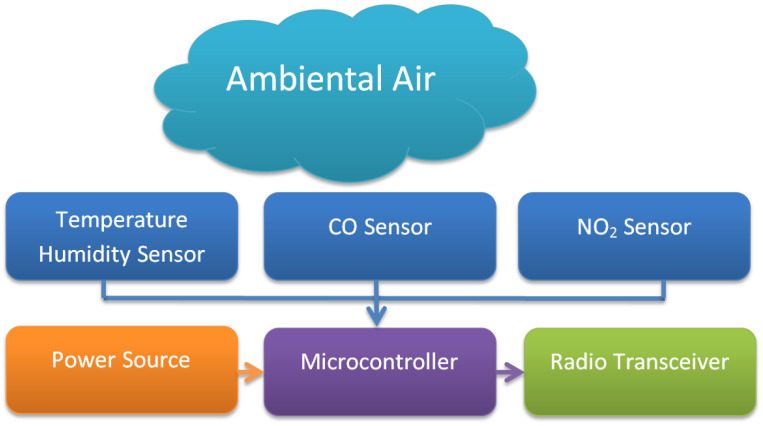
Sensing unit block diagram.

The overall sensing unit architecture is represented in Figure 1 and includes a set of gas sensors used for measuring concentration levels and one separate sensor used for measuring the temperature and air humidity. All are connected to a microcontroller that has a built-in analog-to-digital converter (ADC) used to digitise the incoming sensor readings and then sent to a radio transceiver that ultimately transmits the data to a remote receiving station. The entire sensing unit is powered by a rechargeable battery supply that is integrated into the unit.

Regarding gas sensors, the design makes use of metal oxide semiconductor (MOS) sensors [26] that are relatively cheap, lightweight, and last longer than other sensing technologies. They can be integrated more easily into a design and do not require additional complex circuitry in order to function. A MOS sensor operates on the principle of oxidation or reduction that is induced by the reaction between a specific gas and a heated semiconductor material, thus making the material lose electrons, which consequently decreases its resistance proportional to the level of the gas concentration that has caused the reaction [12]. The use of MOS sensors has its drawbacks because they have low accuracy and can easily be influenced by temperature or wind. However, they are a good solution because of their compactness, are longer-lasting and low-cost, and have traits that enable the creation of a unit packed with multiple sensors, one for each gaseous pollutant that needs to be monitored.

Currently, the sensing unit is equipped with sensors for measuring carbon monoxide (CO), nitrogen dioxide (NO_2_), temperature, and humidity. The sensors are manufactured by AppliedSensor GmbH (currently known as AMS AG). AppliedSensor iAQ-core and AS-MLN use MOS technology in order to measure CO and NO_2_ [27,28].

**Figure 2:**
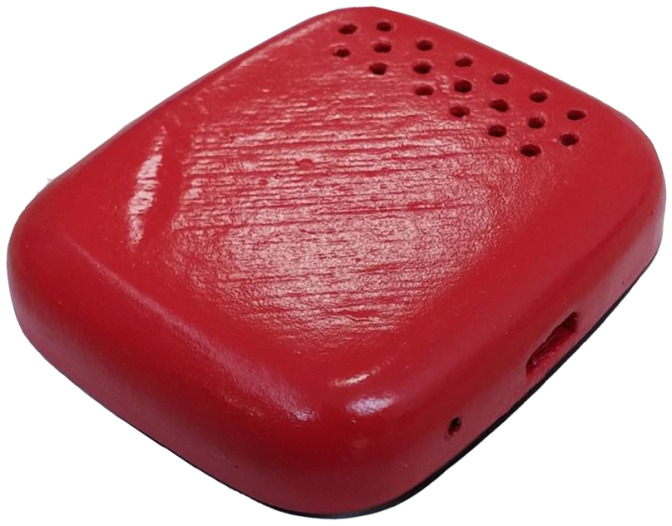
Special mounting unit designed for the sensing unit.

**Figure 3:**
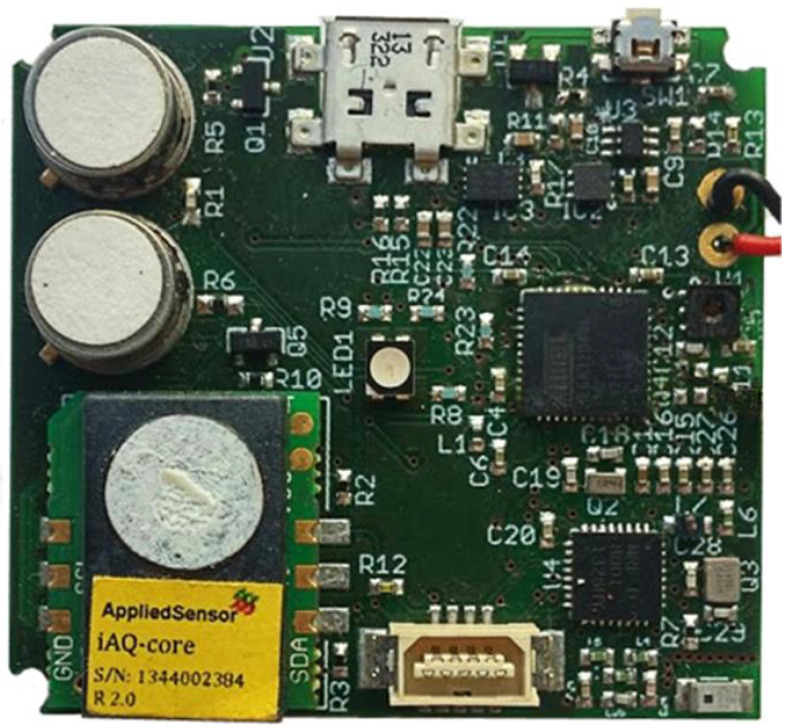
Final sensing unit PCB prototype.

The sensors are housed in a special unit mounted on the top of the UAV above the centre of the chassis, in the payload compartment, safe from any propeller-caused turbulence (Figure 2). Along with the sensors, the microcontroller is integrated inside the sensing unit, on the same printed circuit board (PCB) (Figure 3), together with the rechargeable battery supply and radio transceiver.

We designed an Arduino-compatible board based on the ATmega2560 microcontroller [29] that has 16 analog inputs, 4 UARTs, and 51 digital I/O pins offering plenty of possibilities for future improvements like expanding the number of gas sensors or attaching other additional sensing units that would improve later interpretation of data. The Arduino-compatible platform provides a flexible microcontroller interface with a large community of developers built around it and a considerable large number of open-source libraries, which makes it suitable for rapid prototype implementation.

The gas sensors are connected to the analog pins of the microcontroller, while the temperature and humidity sensor is interfaced using the inter-integrated circuit (I2C) interface, and the radio transmitter is connected using the Universal Asynchronous Receiver/Transmitter (UART) interface. A Digi XBee Pro ZigBee module [30] was chosen as the radio transceiver, with has a purpose of delivering the collected data between the sensing unit and receiving station.

As for the software that runs on the microcontroller, the logic implements a finite state machine that uses the internal ADC to digitise the incoming sensor readings from the NO_2_ and CO sensors and the I2C interface to read data from the temperature and humidity sensor. Then it packs the readings into messages and sends them through the UART interface to the radio transceiver, which finally transmits the readings to a receiving station. All incoming sensor readings are sampled as raw data and packed as comma-separated values (CSV) before being sent to the radio transceiver, which further sends the data in a plain text format, without any additional data encryption, using word length as a reference for any data corruption check at the receiving station.

The main objective of the air quality monitoring system was to perform real-time monitoring of the sensor data, in order to rapidly investigate and mitigate the sources of high pollution in certain areas. The ADC reads sensor values every 1 s, and the data is transmitted to the ground station via the XBee transceiver every 5 s in chunks of 5. The data is then processed on the ground station to produce a point every 15 s in order to eliminate invalid sensor readings. This way, we achieve live monitoring and generate valid points of sensor readings. Real-time monitoring is useful for identifying problems with the data that is being recorded or with the transmission.

An alternative would have been to use a flash drive or any similar on-device persistence solution to store all sensor readings and process them at the end of the flight. This would help us extend the range and cover higher altitudes. However, this would not allow the real-time monitoring of sensor data. Another alternative to the XBee module was to use LoRa modules. LoRaWAN can cover up to 10 km, so it could be used to cover higher altitudes. However, it is suitable for applications that have a low data update rate and can go up to 2–5 min intervals. Thus, it is not appropriate for real-time data monitoring applications [31]. In addition, we are limited by the battery of the multi-copter, which has the autonomy of 50 min, and also by the range of the UAV telemetry link, which is 600 m. In addition, the local legislation does not allow flying drones at high altitudes, practically the range of XBee is larger than the flight limit. Ergo, there is a low benefit added to having a flash drive to store the data or in using LoRa technology.

The entire sensing unit (Figure 3) including the microcontroller, sensors, and radio transceiver, is powered by a rechargeable lithium-polymer battery that includes three cells with a total capacity of 1000 mAh and can supply power for about 90 min of continuous sampling [32]. We use a dedicated battery for the sensing unit in order to have the measurement board completely separate from the multi-copter power system, so the sensor readings will not be affected by the interference from the multi-copter. The power consumed by the frequent sensor readings and RF transmissions is not significant because we are limited by the autonomy of the UAV’s battery. The power consumed by the multi-copter is much larger than the power consumed by the sensing unit.

### 3.2. Multi-Copter

In the last few years, the use of unmanned aerial vehicles has significantly increased because of the advancements in motors, electronics, and microcontrollers that has made possible the integration of previously designed large-scale multi-rotor systems to a smaller scale. In this paper, we propose a pollution measuring system that integrates a multi-gaseous sensing unit with a UAV, and makes use of the spatial exploring capability that a multi-rotor copter can offer.

**Figure 4:**
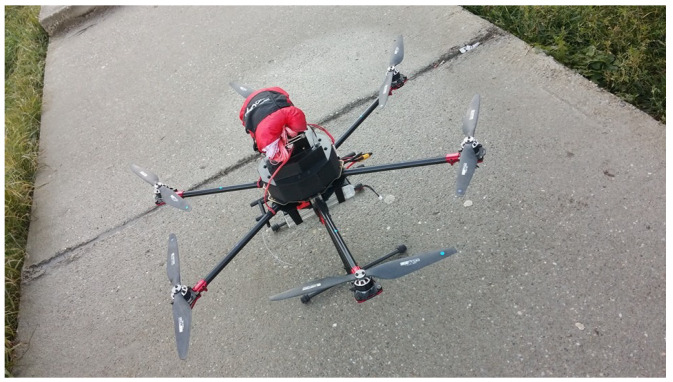
Hexa-rotor aerial copter design.

For the proposed system, an unmanned multi-rotor aerial copter has been assembled using off-the-shelf components (Figure 4). A hexa-rotor aerial vehicle was chosen, which is designed to use six-rotor components, positioned in a six corner star layout, which are attached to a custom made carbon fibre frame, with special mountings to reduce additional rotor vibrations. Compared to a quad-rotor, a hexa-rotor copter makes use of two additional rotors that offer better manoeuvrability and provides the possibility of carrying more payload, but at the expense of a shorter flying time. The rotors are manufactured by KDE-Direct, model KDE2814XF-515KV, and offer an excellent efficiency of 13 g/W with each having attached a 14 × 4.8 carbon fibre propeller manufactured by RC Tiger Motors.

The processing unit consists of an ArduPilotMega (APM) 2.6 board, which is an open-source unmanned aerial vehicle platform particularly designed to control autonomous multi-copters. For navigation, we use a gyroscope, accelerometer, magnetometer, barometer, and GPS. The main reason for having GPS is to allow the drone to navigate autonomously and be able to respect the pre-loaded mission coordinates. Even if the GPS provides some altitude information, it has a very high error. For the altitude, we used the barometer installed on the multi-copter PCB to be able to have accurate altitude measurements, with very low errors.

In the unlikely case of a malfunction while in mid-air, a parachute is integrated into the design as a fail-safe mechanism in order for it to automatically be deployed in case of an emergency. Due to the memory limitations of the APM board, some unused modules were deleted and replaced with the needed features. For the automatic fail-safe mechanism, the auto-deploy libraries were imported from the software API of the Pixhawk Autopilot manufactured by 3D Robotics. Manual parachute deployment has also been implemented using a separate remote controller that connects to the parachute itself and forces the parachute’s release.

The entire flying system, including the six propelling rotors and processing board, is powered by a four-cell rechargeable lithium-polymer battery that has a combined cell capacity of 16,000 mAh. The total weight of the system is approximately 3.9 kg with a total flying autonomy of 50 min and has a total resistance force against the wind of 30 km/h.

The communication with the multi-copter is done using a wireless telemetry link running on 443 MHz, through the integrated module on the APM board. The range of the telemetry link is around 600 m, depending on other radio frequency interference or other wireless devices that might communicate through the same shared medium. The telemetry system is used to control the flight, for example, to update the course of the mission in flight in real-time.

### 3.3. Measurement Methodology

In conducting this research a specific measurement methodology has been developed, which makes use of the flexibility and mobility that a UAV can provide by flying over a certain fixed area. A flying mission represents deploying the multi-copter to follow a predefined flying course, during which it collects and sends the sensed data. This enables the recording of pollution information from the gas sensors that are attached to the sensing unit, and the interpretation of that data at the receiving station.

We used two types of flying missions in order to evaluate the proposed system. The first type of flying mission is an altitude mission, which is simply a continuous slow increase in altitude up to the desired height following a straight altitude path from the ground up, by taking advantage of the UAV characteristic of stability and very little influence from any wind turbulence. Throughout the ascension, data is sampled and recorded, therefore allowing for interpretation of how gaseous concentration disperses with altitude.

**Figure 5:**
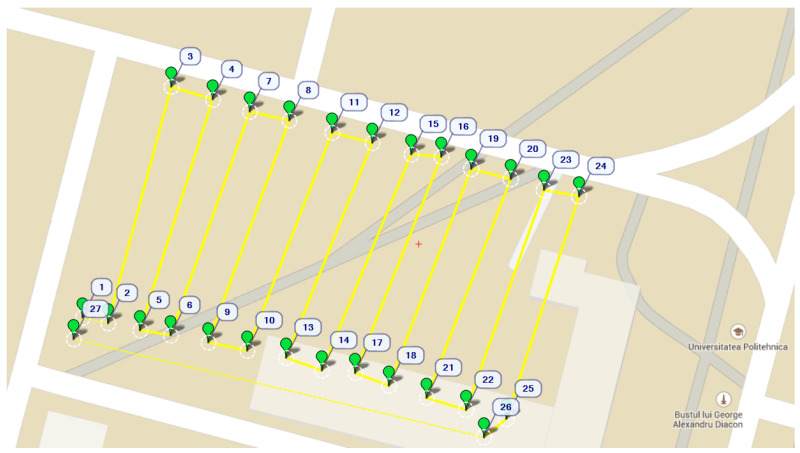
Mission course.

In the second type of flying mission, the UAV completes a marked course (represented in Figure 5) at a given constant altitude and continuously samples data, in order to generate fine-grained pollution heat maps that can estimate the dispersion of a specific gaseous pollutant over a certain area.

## 4. Evaluation and Results

This section presents the analysis of sensor readings that were obtained during the evaluation of the proposed system. The experiment was conducted in multiple urban environments and on the outskirts of Bucharest, which is the largest metropolitan city in Romania.

The data collection was made throughout the autumn and winter, when temperatures varied from −5 to 10 °C and the humidity fluctuated depending on the weather conditions, with some of the measurements taken during snowy days. The graphics include readings from four types of sensors: Temperature, humidity, nitrogen dioxide (NO_2_), and carbon monoxide (CO).

### 4.1. Air Quality Reference Indices

Multiple studies have been conducted regarding the effects that various concentrations of gaseous pollutants have on human health [33,34,35,36]. In this paper, we consider two gaseous pollutants: CO and NO_2_. CO is a toxic gas that affects the respiratory and cardiovascular systems and reduces the capacity to transport oxygen through the body. It may also cause fatigue, irritability, migraines, nausea, dizziness, and lack of coordination [37]. NO_2_ is a toxic gas that affects the lung tissue and may cause pulmonary diseases and respiratory difficulties [38].

The CO and NO_2_ exposure limits, as provided by the Romanian Ministry of Environmental Protection [39] are presented in Table 1 and Table 2. We consider them as reference values for our experiments. The current law in Romania states that the limit value for CO, for protecting human health, is 10 $\mathrm{mg}/m^{3}$ [37]. This is computed as the maximum value of the average for 8 h. As for NO_2_, the current law states that the annual critical level for vegetation protection is 30 $\mu g/m^{3}$, the annual limit level for human health protection is 40 $\mu g/m^{3}$, and the hourly limit value for human health protection is 200 $\mu g/m^{3}$. The alert threshold is 400 $\mu g/m^{3}$ and it is measured during 3 h in certain locations over at least 100 ${\mathrm{km}}^{2}$ [38]. These thresholds and ranges may be different from one country to another [40], but their meaning is similar.

### 4.2. University Area

**Figure 6:**
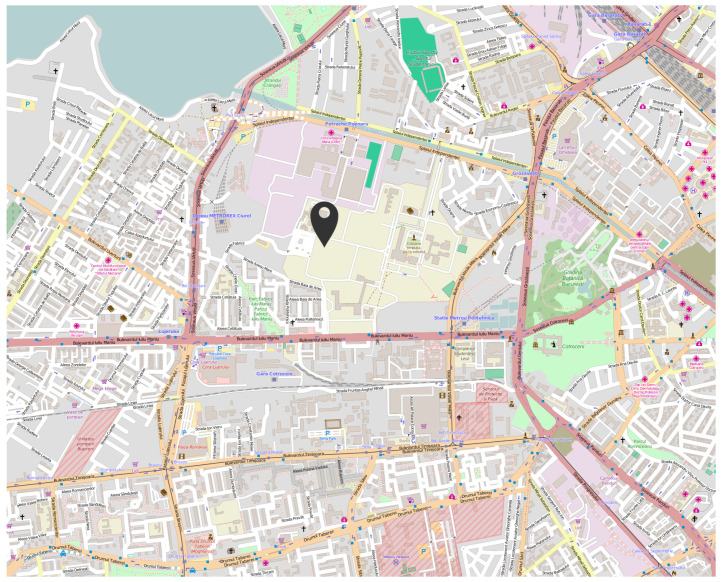
University Politehnica of Bucharest campus, location of the first urban area sensing mission.

**Figure 7:**
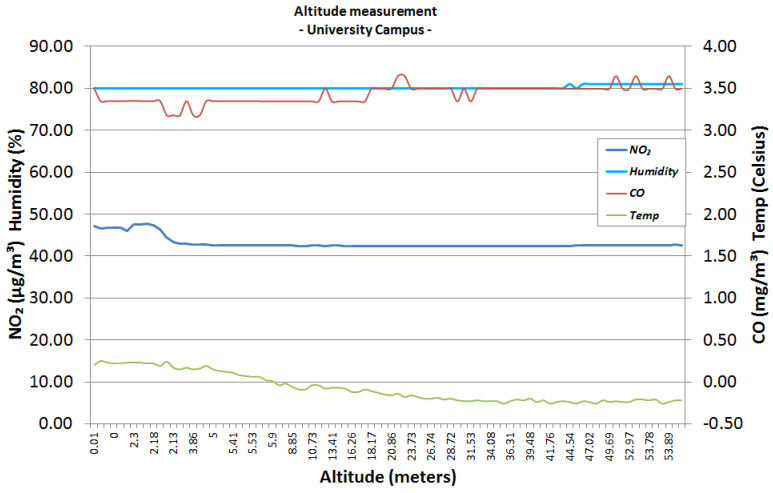
University area, snowy day, winter, 50 m altitude.

**Figure 8:**
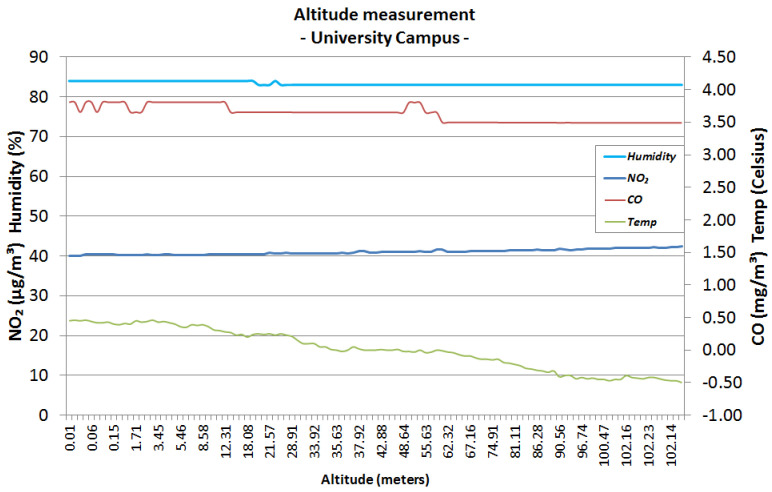
University area, snowy day, winter, 100 m altitude.

**Figure 9:**
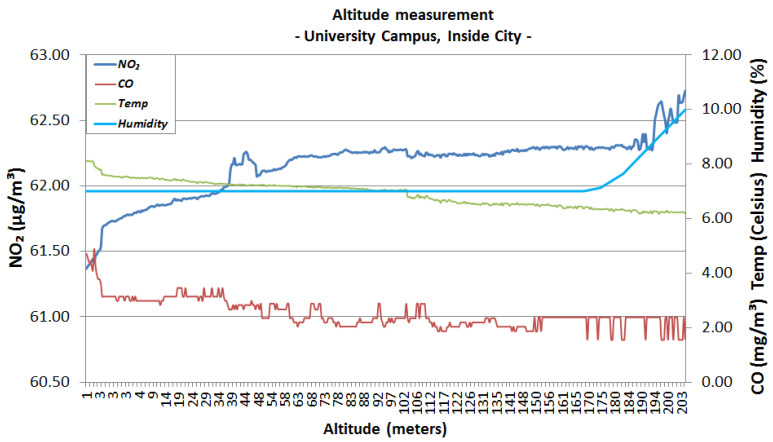
University area, sunny day, autumn, 200 m altitude.

**Figure 10:**
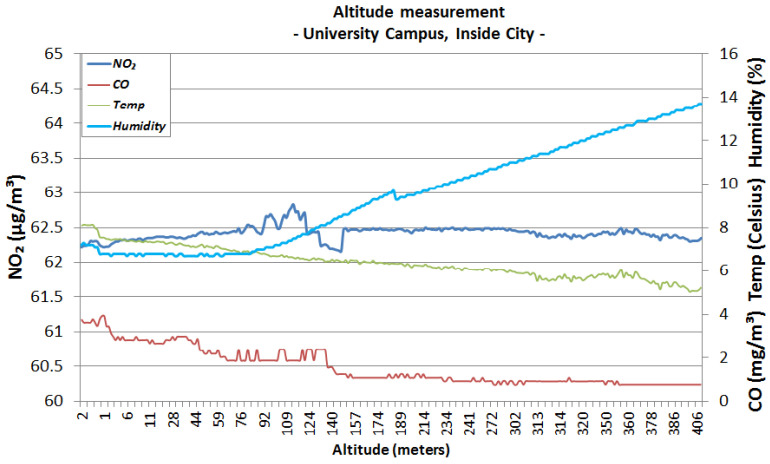
University area, sunny day, autumn, 400 m altitude.

The first urban area sensing mission was performed in the campus of the University Politehnica of Bucharest (Figure 6), both in winter conditions (snowy day) and autumn conditions (sunny day). The following graphics represent the measurements made using the altitude method that has been described in the previous section. Figure 7 and Figure 8 analyse the change in data measured by sensors in respect to altitude during fair winter weather conditions (up to 100 m altitude) while Figure 9 and Figure 10 analyse the data collected during sunny autumn weather conditions (up to 400 m altitude). Both scenarios were recorded during fair wind speeds of 0 to 2 km/h, with the humidity generally stable at a low almost constant value, and the pressure at a normal value of 760 mmHg (1 atm).

As observed in Figure 7 and Figure 8, the level of NO_2_ remains stable during the entire ascension of the hexa-copter from ground level to the designated altitude, with a value between 40 and 50 $\mu g/m^{3}$, which is within the Excellent range based on the gradient values presented in Table 2. Analysing the CO levels, the values tend to stabilise between 3.2 and 3.8 $\mathrm{mg}/m^{3}$, which are classified as Very Good based on Table 1.

Figure 9 and Figure 10 show the results from the sunny autumn days. Analysing the variations, it appears that the level of NO_2_ has increased with respect to altitude from 61.40 $\mu g/m^{3}$ at ground level to 62.70 $\mu g/m^{3}$ at 200 m of altitude and tends to stabilise to a value of 62.40 $\mu g/m^{3}$ throughout the ascent to 400 m. These levels still qualify within the Very Good range. On the other hand, the CO concentration tends to decrease with altitude, dropping from a 5 $\mathrm{mg}/m^{3}$ at ground level to a 2 $\mathrm{mg}/m^{3}$ value at 200 m and continuing to fall to around 1 $\mathrm{mg}/m^{3}$ at 400 m of altitude. The values measured at lower altitudes qualify as Very Good, while the increase in altitude offers Excellent levels of concentration. The values were consistent throughout other days with similar weather conditions.

**Figure 11:**
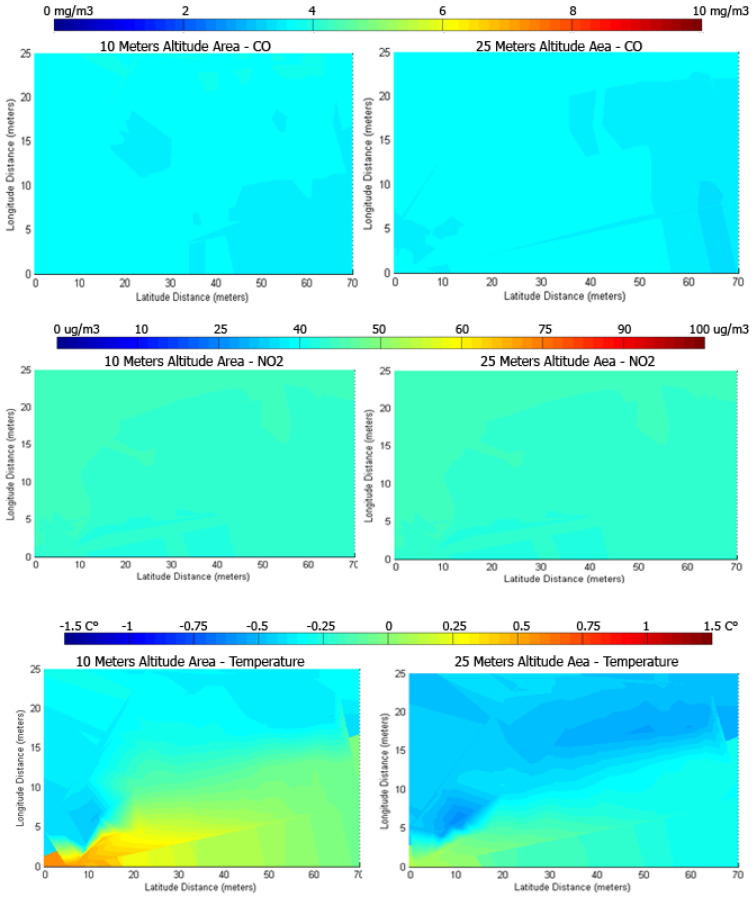
Heat-maps representing changes in gaseous concentrations and temperature for the mission conducted in the winter at the university campus.

Figure 11 includes heat-maps that represent an illustration of the measurements gathered during the winter snowy day mission, conducted in the university campus. The measurements are taken by using the second type of flying mission (described in Section 3.3), at a constant altitude: One mission to measure at 10 m and another one to measure at 25 m. The 10-m and 25-m CO heat maps show little variation between each other, with values ranging from 3.4 to 4 $\mathrm{mg}/m^{3}$, which is a Very Good value in the gradient. The NO_2_ heat maps also show little variation, with levels ranging between 40 to 48 $\mu g/m^{3}$. The temperature variation is just 2 °C.

### 4.3. City Peripheral Residential Zone

**Figure 12:**
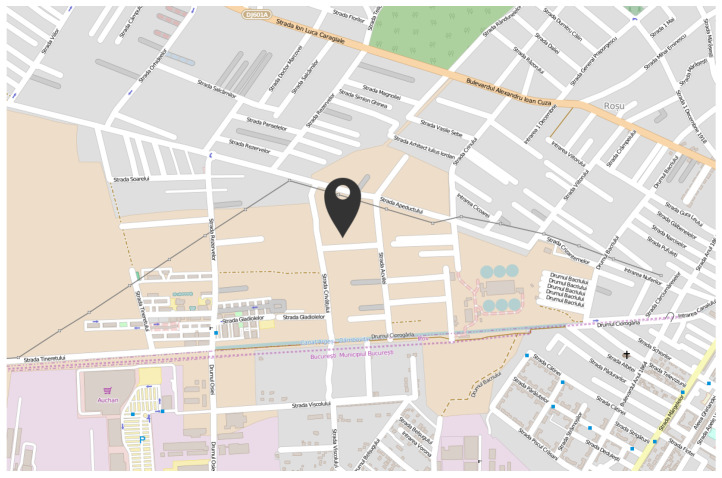
Roșu Town, location for the peripheral area mission.

In the second scenario, the measurements were conducted in the suburbs of the city, as pictured in Figure 12, in a residential housing area near the ring road of the city. This road is a high-speed driveway, mostly circulated by heavy vehicles 24 h a day. Another noteworthy observation is that the area is located near one of the main highly circulated motorways, a waste incinerator, and a cesspool, all of which contribute to the air pollution in the area.

**Figure 13:**
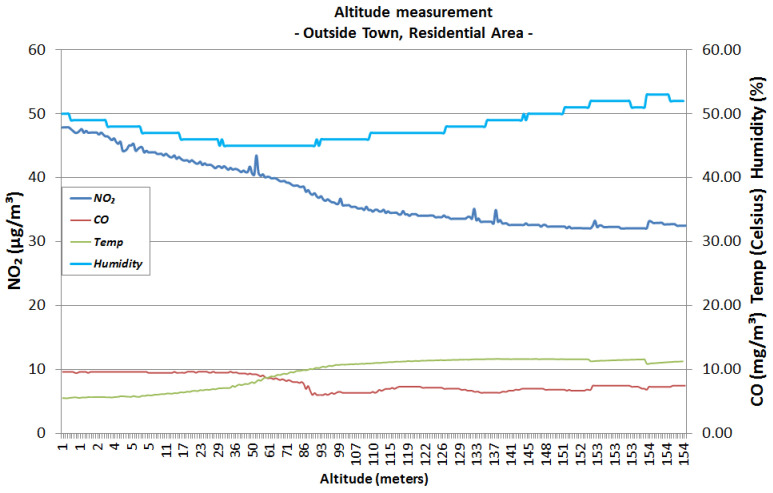
City peripheral residential zone, from ground level to 150 m in altitude.

In Figure 13, the sensor readings show that the level of NO_2_ emissions starts from 49 $\mu g/m^{3}$ at ground level and gradually decreases to 32 $\mu g/m^{3}$ at 150 m of altitude. Looking at the reference indices for NO_2_ presented in Table 2, this peripheral side of the city is at the limit of Excellent and Very Good, similar to the previously measured data at the university campus. By analysing the CO emissions, it can be observed that at ground level there is a concentration of 10 $\mathrm{mg}/m^{3}$ and it decreases to 8 $\mathrm{mg}/m^{3}$ at 150 m of altitude, which is at the limit between Medium to Bad, according to the indices in Table 1. The current legislation states that 10 $\mathrm{mg}/m^{3}$ is the limit for protecting human health, so CO should be constantly monitored in this area to detect when it goes beyond the legal limit.

**Figure 14:**
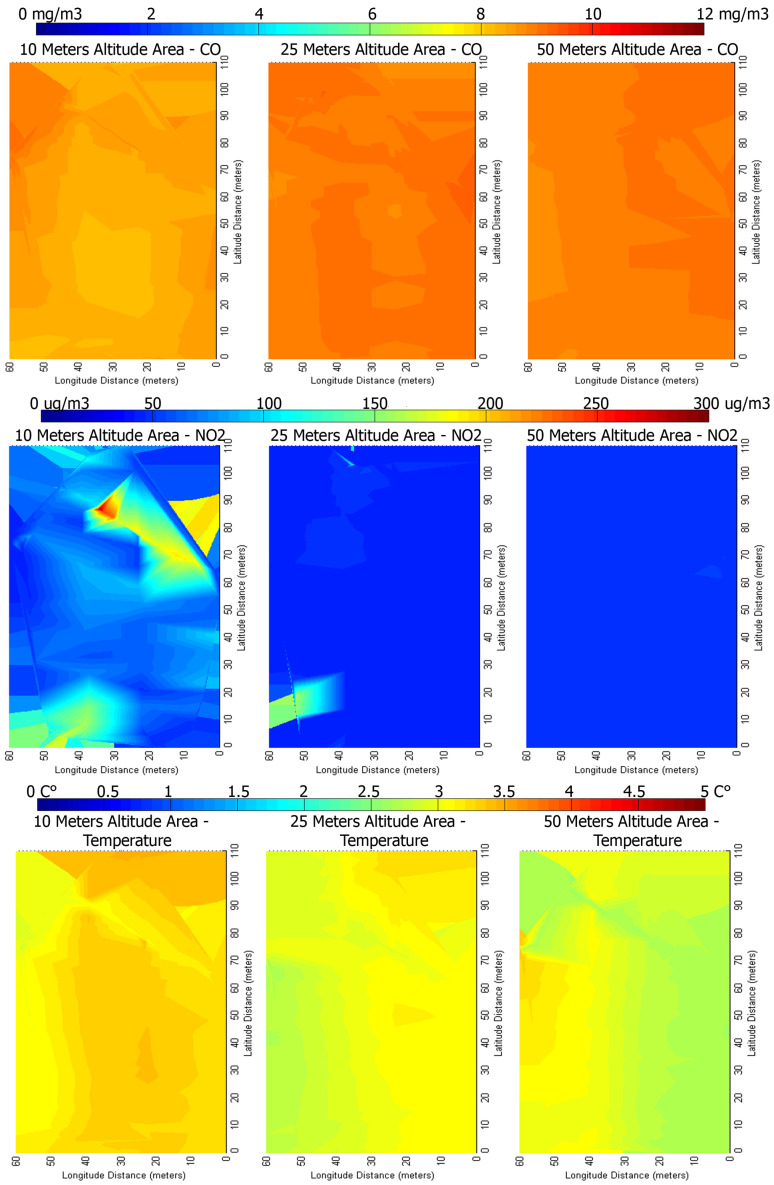
Heat-maps representing changes in concentration for the mission conducted on the outskirts of the city of Bucharest.

Figure 14 includes heat-maps that represent an illustration of the measurements gathered in the city peripheral residential area. The measurements are taken by using the second type of flying mission (described in Section 3.3), at constant altitude: One mission to measure at 10 m, another mission to measure at 25 m, and the last one to measure at 50 m. The CO concentration has a small variation, between 7 $\mathrm{mg}/m^{3}$ (Medium) to 11 $\mathrm{mg}/m^{3}$ (Bad), while the NO_2_ has a more interesting behaviour: At 10 m altitude it is mostly stable between 47–48 $\mu g/m^{3}$ (Excellent), while rapidly increases up to 170 $\mu g/m^{3}$ (Medium) but only in a particular area, and up to 110 $\mu g/m^{3}$ (Good) in another. The temperature variation is between 2 and 4 °C.

### 4.4. City Mall Underground Parking

In the third scenario, the measurements were conducted in the parking lot of a city mall during evening rush hours, with the parking lot occupying around 40% and more than half of the cars only just arriving moments before the mission began. The data were recorded throughout the whole parking lot, during two time intervals, at a constant altitude of 1.5 m.

**Figure 15:**
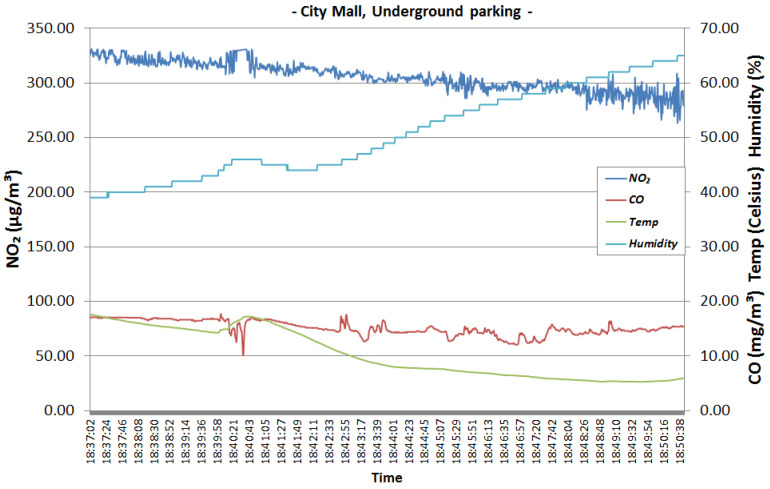
City mall underground parking—first time interval.

**Figure 16:**
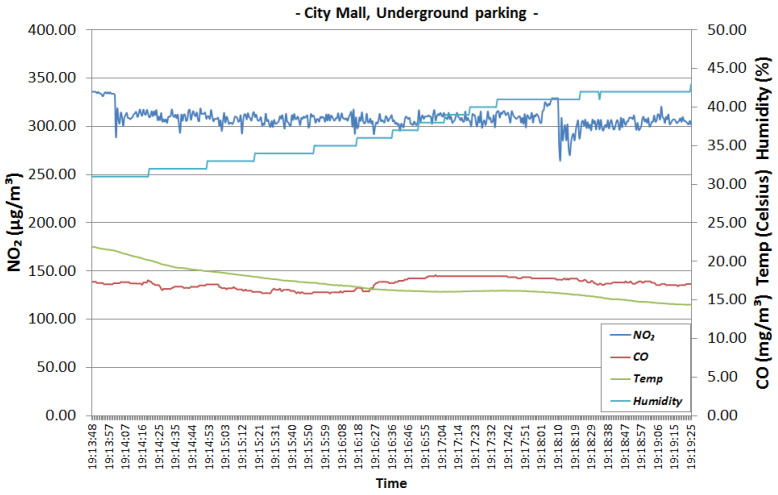
City mall underground parking—second time interval.

Figure 15 and Figure 16 represent the readings collected in the parking lot. The two graphics show that the levels of NO_2_ and CO emissions are high. NO_2_ is somewhere between 250 to 340 $\mu g/m^{3}$ which is considered Bad on the gradient scale presented in Table 2, while the CO concentration is between 10 to 20 $\mathrm{mg}/m^{3}$, which is considered Very Bad. Both NO_2_ and CO concentrations exceed the legal limit for protecting human health. Most of the gaseous pollutants are emanating from the incomplete combustion of gasoline in car engines. A person cannot spend too much time in an area with so much pollution without health-related implications.

We analyse how sensor data changes throughout time in order to see how gases or air properties can change and affect each other. For example, we can observe that when temperature drops, humidity increases (Figure 15 and Figure 16). Considering the high speed of air currents in the parking lot and the continuous movement of cars and people, we were expecting to see some variation in the NO_2_ and CO concentrations. However, we can see that they are approximately constant, and there is no relation between each other or between them and the air properties.

### 4.5. Discussion

We evaluated the proposed system by collecting data in different parts of a metropolitan large city, during two seasons, and creating the vertical profiles and heat-maps of pollutants. On the university campus, the pollution level varies from Very Good to Excellent. In the city peripheral residential area, NO_2_ varies between Very Good and Excellent and CO varies between Bad and Medium. CO should be constantly monitored in this area to detect when it goes beyond the legal limit. Pollution reaches critical levels in the underground parking lot, where NO_2_ is in the Bad range and CO is in the Very Bad range. Both NO_2_ and CO concentrations exceed the legal limit for protecting human health. A human can spend a short amount of time in an underground city parking lot before it starts affecting one’s health.

Regarding the concentration of the two gaseous pollutants that have been studied in this article, the CO is present in a higher concentration than NO_2_, which brings the air quality close to a level categorised as Bad in some areas. We determined that the outskirts of the city are more polluted than the city centre because of the factories and waste collecting centres that raise the level of CO in the air. NO_2_ does not fluctuate considerably in the city centre, but in the city suburbs, its value goes down with respect to altitude. As for the CO, its level also becomes lower with the altitude, but at a lower rate. These results are compatible with the ones presented in the related work.

## 5. Conclusions

UAVs are now capable of advanced autonomous missions in many fields of study involving surveillance or remote sensing on land, ocean, or hostile environments. In recent years, researchers have focused their attention on monitoring air pollution through UAVs [25]. In this paper, we proposed a UAV-based air quality real-time monitoring system that provides insight regarding how pollution acts at various altitudes. We implemented a low-cost solution that is able to measure pollution in large areas and at high altitudes (even above average buildings) and can be used to generate fine-grained heat-map representations for specific locations. We evaluated our system in various locations throughout a metropolitan city, during different seasons, and generated vertical profiles and fine-grained heat-maps that display the level of pollution of specific areas based on different altitudes. We found that some areas need constant air quality monitoring in order to detect when pollution goes beyond the legal limit for protecting human health. The proposed UAV-based pollution measurement system can be improved in future studies to better estimate the dispersion of gaseous pollutants in a three-dimensional space. We also aim to include other pollution sensors and perform the vertical profiles and fine-grained maps of those pollutants.

## Figures and Tables

**Table 1:** CO exposure limits.

Level	Concentration ($\mathrm{mg}/m^{3}$)	Concentration (ppm)
Excellent	0–2.(9)	0–2.61(9)
Very Good	3–4.(9)	2.62–4.366(9)
Good	5–6.(9)	4.367–6.113(9)
Medium	7–9.(9)	6.114–8.733(9)
Bad	10–14.(9)	8.734–13.0(9)
Very Bad	>15	>13.1

**Table 2:** NO_2_ exposure limits.

Level	Concentration ($\mu g/m^{3}$)	Concentration (ppb)
Excellent	0–49.(9)	0–25.24(9)
Very Good	50–99.(9)	25.25–50.50(9)
Good	100–139.(9)	50.51–70.70(9)
Medium	140–199.(9)	70.71–100.(9)
Bad	200–399.(9)	101–201.(9)
Very Bad	>400	>202

## Data Availability

Not applicable.
